# Emel Hospital, Syria

**Published:** 2017-02-10

The civil war in Syria is arguably the worst humanitarian catastrophe since the Second World War. According to recent sources, over 250,000 have been killed, the same number wounded or missing and over half of the country's population of 22 million having been displaced from their homes, with 3.8 million being made refugees.

Aleppo, in the north of the country, has received far more than its fair share of mass destruction and has been the worst hit city in the civil war. It has seven remaining functioning hospitals, but supplies and medical care is dwindling. Several hospitals have been directly hit by bombing on more than one occasion and refugees head out of the city for medical treatment. One of the hospitals providing emergency care, including eye care, is Emil Hospital – located 70km from the centre of Aleppo. Emel Hospital provides most of the surgical services in the area, treating many severe injuries resulting from the violence. It is one of 42 similar field hospitals inside Syria, 65% of which have suffered attacks.

The medical director of Emel Hospital is Ahmed Hassan Batal, a paediatric ophthalmologist from Saudi Arabia. Since the conflict began, he and his team of 10 doctors and 20 nurses have performed more than 9,000 complex surgical procedures on patients with horrific injuries caused by the violence. The medical facilities at Emel are barely adequate: much of the equipment is secondhand, having been donated from several sources, and there is a huge shortage of drugs and dressings.

Thankfully, Emel Hospital has, at the time of writing, been spared the bombing that many other hospitals have endured. A neighbouring hospital, only one kilometer away, has been bombed twice. One can only imagine the anxieties of those working at Emel that it may suffer the same fate. However, Dr Batal describes the morale of the medical staff as remaining “very good”. He stresses the fact that staff members ignore the risks of working at the hospital for the benefit of all patients, whatever their beliefs and politics may be. Dr Batal himself has committed to working at the hospital until the conflict ends.

Despite the daily challenges of working at Emel, Dr Batel has remained an active member of the Examinations Committee of the International Council of Ophthalmology (ICO) – an international organisation which represents and serves professional associations of ophthalmologists. In his role, Dr Batal reviews all ICO examination papers, sets appropriate questions, and ensures the validity, accuracy and standardisation of each examination paper. He has also agreed to pay the examination fees of all Syrian ophthalmologists wishing to take ICO examinations and has pledged to continue doing so until the conflict in Syria ends – thereby ensuring that Syrian ophthalmologists are not left behind in their professional development as a result of the conflict. As a result, 62 Syrian ophthalmologists have already sat the ICO examinations in Damascus and many more wish to take the examinations in 2017.

I would like to thank Dr Batal on behalf of the ICO and the ICO Examinations Committee for his kindness and humanity. He is an example to us all.

**Simon Keightley FRCS FRCOphth**

Director for Examinations

International Council of Ophthalmology

*Emel Hospital is in need of support. If you can help, please contact Simon Keightley via email: s.keightley@virgin.net*

**Figure:**
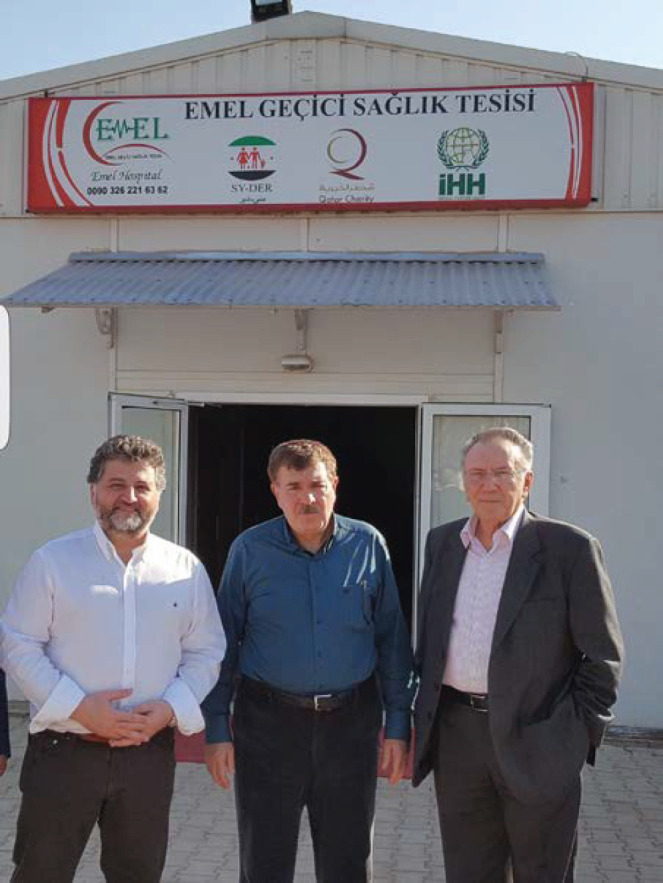
Dr Ahmed Batal (left) at the entrance to Emel Field Hospital. In the centre is Dr Hamedy Osman, the founder of the hospital. Next to him is Dr Nabil Mureden, a volunteer surgeon and chairman of the Italian-Syrian community in Italy

**Figure:**
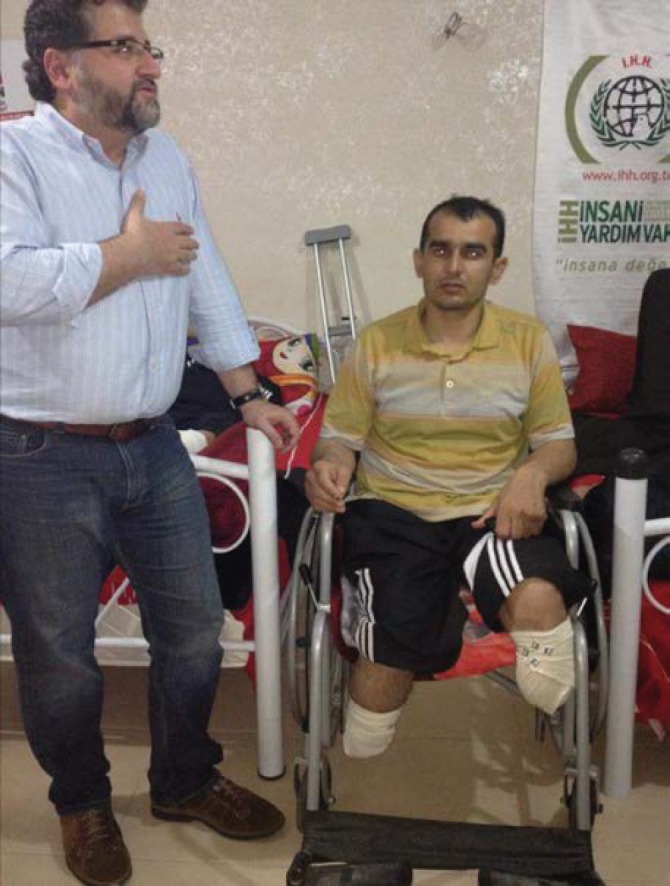
Dr Batal with patient with bilateral lower limb amputations

**Figure:**
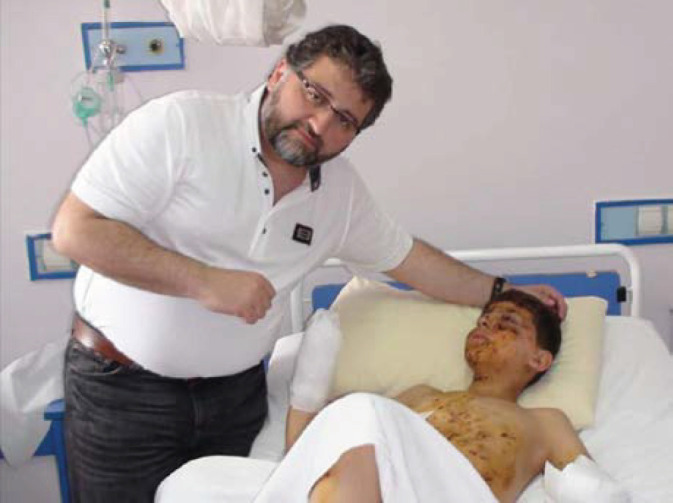
Dr Batal with a victim of the conflict

**Figure:**
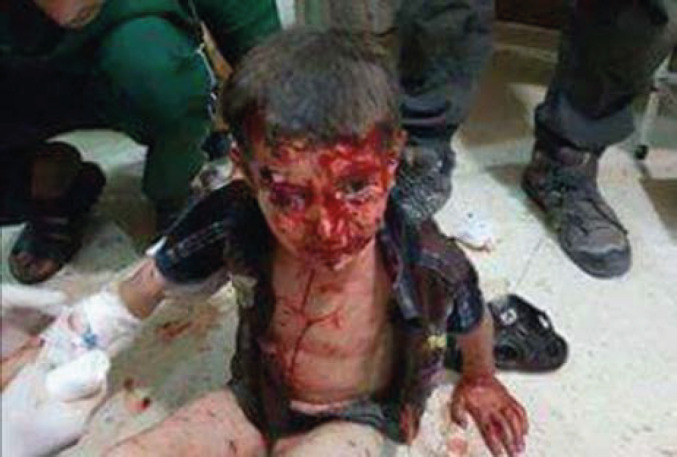
Child victim of the conflict at Emel Hospital

**Figure:**
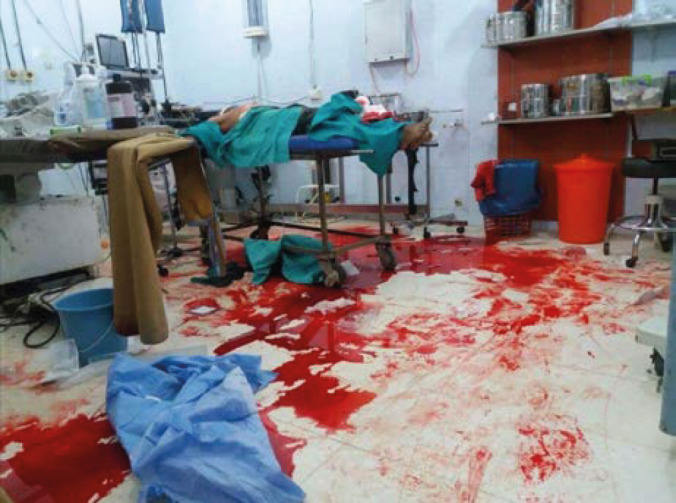
Operating theatre following surgery involving a severe trauma case

**Figure:**
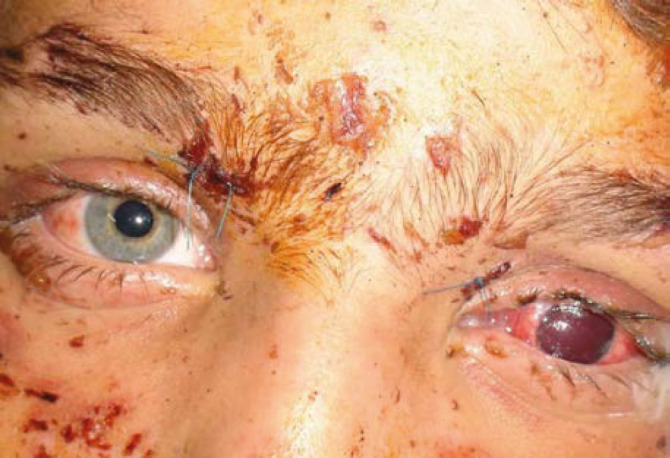
Severe left eye injury following trauma

